# Correlation between clinical variables and overall survival time in dogs with multicentric B-cell lymphoma: a retrospective study in Taiwan

**DOI:** 10.3389/fvets.2026.1870235

**Published:** 2026-08-17

**Authors:** Chiao-Hsu Ke, Shang-Lin Wang, Wei-Hsiang Huang, Ka-Mei Sio, Jih-Jong Lee, Yuan-Yuan Xia, Chen-Si Lin

**Affiliations:** 1 Department of Veterinary Medicine, School of Veterinary Medicine, National Taiwan University, Taipei, Taiwan; 2 Graduate Institute of Veterinary Clinical Science, School of Veterinary Medicine, National Taiwan University, Taipei, Taiwan; 3 Animal Cancer Center, College of Bioresources and Agriculture, National Taiwan University, Taipei, Taiwan; 4 National Taiwan University Veterinary Hospital, College of Bioresources and Agriculture, National Taiwan University, Taipei, Taiwan; 5 Graduate Institute of Molecular and Comparative Pathobiology, School of Veterinary Medicine, National Taiwan University, Taipei, Taiwan

**Keywords:** cancer epidemiology, canine multicentric B-cell lymphoma, flow cytometry, immunophenotyping, PCR for antigen receptor rearrangement

## Abstract

**Introduction:**

Multicentric B-cell lymphoma (MBCL) is one of the most commonly diagnosed spontaneous hematopoietic malignancies in dogs. However, few studies have characterized dogs with MBCL in Taiwan, and the prognostic value of clinical variables, including clinical substage and treatment response, remains under investigation. This study aimed to characterize canine MBCL in Taiwan and evaluate associations between selected clinical variables and overall survival time (OST).

**Methods:**

Medical records of 441 dogs diagnosed with lymphoma were retrospectively reviewed. Cases with peripheral lymph node enlargement and uniformly large neoplastic cells on cytology and/or histopathology were included. Immunophenotypes were primarily determined by flow cytometry, with PCR for antigen receptor rearrangement used in a small subset for clonality assessment. Dogs that completed at least one full cycle of CHOP chemotherapy were included in the OST analysis and stratified by clinical variables.

**Results:**

Dogs with clinical signs (substage b) had shorter OST (median, 140 days; 95% CI, 126–199) than asymptomatic dogs (substage a: median, 186 days; 95% CI, 164–248). Dogs achieving complete or partial response to CHOP had longer OST (median, 196 days; 95% CI, 151–239) than dogs with stable or progressive disease (median, 182 days; 95% CI, 140–196). No significant differences in OST were observed according to serum calcium concentration (>10.5 mg/dL) or advanced clinical stage (IV–V). Cox proportional hazards analyses indicated that clinical signs (HR, 2.27; 95% CI, 1.04–4.96; *p* = 0.040) and failure to achieve an objective response (HR, 2.09; 95% CI, 1.06–4.16; *p* = 0.035) were associated with increased mortality.

**Discussion:**

This study provides the first epidemiological and immunophenotypic characterization of canine MBCL in Taiwan. Clinical substage and initial response to CHOP were associated with OST, although further prospective studies are needed to validate these findings and more comprehensively characterize canine lymphoma in Taiwan.

## 1 Introduction

Canine lymphoma is one of the most commonly diagnosed spontaneous hematopoietic malignancies (1). The lymphoma diagnosis is based on cytology or histopathological examinations of the aberrant lymph nodes, blood, or bone marrow (2). A complete medical history, comprehensive clinical evaluations, and additional tests are also usually taken into consideration (3). For a detailed diagnosis of lymphoma, flow cytometry (FC) and PCR for antigen receptor rearrangements (PARR) are effective technologies in determining immunophenotypes (4). The significance of immunophenotyping is that it appears to correspond with prognosis. Dogs with B-cell lymphoma (BCL) are more responsive to chemotherapy, leading to a better prognosis. However, dogs with T-cell lymphoma (TCL) tend to be more resistant to chemotherapy drugs and thus have decreased overall survival time (OST) (5). Surgical resection followed by histopathology and immunohistochemistry (IHC) staining provides valuable information for the morphological and phenotypic diagnosis of lymphoma (6). In recent years, owing to their convenience, FC and PARR have become two of the most common diagnostic tools for immunophenotyping because these assays can be performed in cells obtained via fine-needle aspiration (FNA) of an enlarged lymph node (2). FC analysis can simultaneously identify lymphoid markers expressed on the cells to distinguish T or B cell subpopulations according to the multiple fluorescence-conjugated antibodies (7). Phenotyping using multiple cell markers has been widely used to diagnose several subtypes of lymphoma with high sensitivity (3, 4, 7–9). Therefore, FC is often the first-line choice for diagnosis. In contrast to FC, PARR assesses clonality in a population of lymphoid cells by amplifying the variable regions of immunoglobulin (for B cells) or T cell receptors (TCR) (10, 11). The neoplastic cells descend from a single clone and thus express receptor genes with the same sequence and size (12). Although one previous study comparing PARR with FC reported that the sensitivity of PARR assay is lower than that of FC, PARR is the only assay that can illustrate the true clonality of lymphoid cells (4).

Diffuse large B-cell lymphoma (DLBCL) is the most common lymphoma type in dogs, accounting for 50–60% of all canine lymphoid neoplasms. Canine DLBCL requires a comprehensive approach that combines clinical evaluation, cytology, and immunophenotyping to ensure accurate identification of the disease. Dogs affected by DLBCL usually show moderate to severe enlargement of peripheral lymph nodes (13). Cytological examination allows for initial identification and characterization of neoplastic cells, which is essential for diagnosis (14, 15). Therefore, in dogs with DLBCL, peripheral lymphadenopathy with peripheral lymphocytosis is usually identified in physical and cytological examinations (16, 17). To accurately determine the immunophenotypes of neoplastic cells, FC and/or PARR assays have been utilized (4). Several prognostic factors have also been reported in dogs with DLBCL. Several of them, including reproductive status, anemia, and neutrophil-to-lymphocyte ratio, significantly impact survival outcomes (18). Another study identified a 6-gene signature (*IL2RB*, *BCL6*, *TXK*, *C2*, *CDKN2B*, and *ITK*) strongly associated with lymphoma-specific survival (19), highlighting the prediction outcome in dogs with DLBCL with immune-related gene signatures. MicroRNA profiles have also been correlated with disease progress; it has been reported that miR-192-5p and miR-16-5p expression were significantly downregulated in dogs with increased progression-free survival (PFS) (20). However, because histopathological and IHC confirmation was not available for all cases in the present retrospective cohort, the current study uses the broader term MBCL rather than DLBCL for study-specific cases.

Although several publications have reported the outcomes of dogs categorized by several clinical variables (6, 21), few investigations have provided a characterization of dogs with lymphoma in Taiwan. Furthermore, several prognostic biomarkers, such as immune landscapes, microRNA, immunophenotypes, and morphological diagnosis, have been proposed. However, the prognostic values of clinical variables, including clinical substage and treatment responses, in canine MBCL remain controversial and are still under investigation. Compared with other prognostic biomarkers, this clinical evidence can directly provide first-line prognostic value to veterinarians and owners. Therefore, this study aimed to investigate the correlation between OST and different clinical parameters of dogs with MBCL. Through the current study, we hope to provide more valuable prognostic information for dogs with MBCL in veterinary clinical medicine.

## 2 Materials and methods

### 2.1 Patient selection and evaluation

**Figure 1:**
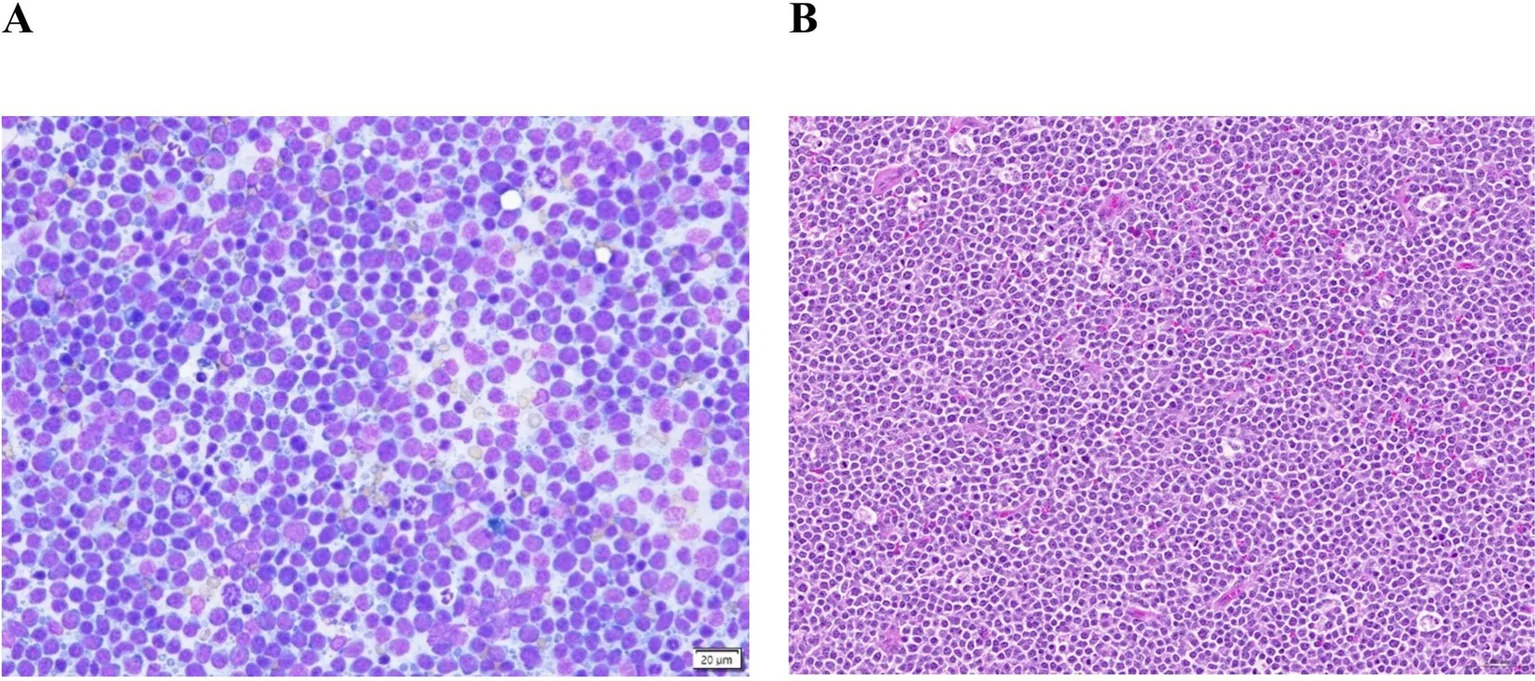
Cytological and histopathological evaluation of samples affected by MBCL. **(A)** Fine needle aspiration of lymph node. Neoplastic lymphocytes are intermediate to large (more than 2–3 times the size of red blood cells). **(B)** Representative histopathological images of samples diagnosed as MBCL. The neoplastic lymphocytes have a high nuclear-to-cytoplasmic ratio and round nuclei with coarse chromatin. The nuclei are 2–2.5 times the diameter of erythrocytes. MBCL, multicentric B-cell lymphoma.

Dogs diagnosed with MBCL at National Taiwan University Veterinary Hospital (NTUVH), Taipei, Taiwan, between 2013 and 2026 were evaluated in this study. Cases were clinically and cytologically and/or histopathologically assessed, and B-cell lineage was determined by FC and/or PARR. Because histopathological assessment and IHC were not available for all cases, cases were not uniformly classified as DLBCL according to WHO histological criteria. Cases were included if they presented with generalized peripheral lymphadenopathy and uniformly large neoplastic cells on cytological and/or histopathological evaluation (Figure 1), regardless of the presence of peripheral lymphocytosis, and were then further immunophenotyped via FC and/or PARR. In a minor subset of patients where samples were insufficient or lacked viability for FC, PARR was utilized for initial classification. However, these PARR-only cases were classified as presumptive TCL and were restricted to descriptive epidemiological reporting and were excluded from survival analyses to prevent bias due to lineage misclassification. Medical records were also carefully reviewed. Breed, sex, neutering status, age at diagnosis, clinical signs, complete blood count (CBC), serum biochemistry profile, diagnostic procedures, clinical stage according to the WHO clinical staging system, treatment responses, and overall survival were retrospectively analyzed from medical records. Furthermore, dogs were subclassified into substage a (absence of systemic signs) or substage b (presence of systemic signs). In this study, the clinical signs utilized to classify a patient as substage b included systemic manifestations such as notable weight loss, loss of appetite (anorexia), severe lethargy, fever, vomiting, and/or diarrhea documented in the medical records. Hematologic abnormalities alone were not used to define substage b. For the evaluation of calcium levels, uncorrected total serum calcium concentrations obtained before the initiation of CHOP chemotherapy were utilized. Patients with obvious non-neoplastic causes of elevated calcium, such as severe chronic kidney disease or primary hyperparathyroidism, were excluded from the calcium analysis. To evaluate the relative prognostic impact of calcium levels and maintain statistical power, the continuous variable of total serum calcium was dichotomized at the median value of the cohort (10.5 mg/dL), classifying patients into higher (> 10.5 mg/dL) and lower (≤ 10.5 mg/dL) serum calcium groups.

Canine lymphoma patients that received the standard CHOP chemotherapeutic protocol were further analyzed for OST. The options included 25- (22), 19- (23), and 15- (24) week protocols, which were determined by attending clinician preference. Dogs with other medical interventions were excluded from the OST analysis. The therapeutic responses were determined using the Veterinary Cooperative Oncology Group response evaluation criteria for lymphoma (25). Response to the initial CHOP protocol was determined retrospectively by a review of medical records. A complete response (CR) was defined as the complete disappearance of all clinically measurable diseases. Partial response (PR) was defined as a reduction (above 30% but below 100%) in the mean sum longest diameter of measurable lesions and no new lesions being recognized. Target lesions compared to baseline with a < 20% increase or <30% decrease were characterized as stable disease (SD); however, an increase in target lesions of >20% or the occurrence of new lesions was classified as progressive disease (PD). Only dogs that completed at least one full cycle of CHOP chemotherapy were included in the OST analysis. In this study, one full cycle was operationally defined as completion of the first complete CHOP drug sequence, including vincristine, cyclophosphamide, doxorubicin, and prednisolone/prednisone, according to the attending clinician’s selected schedule. OST was defined as the time between the initiation of treatment and death. In the survival analysis, dogs were censored if they were lost to follow-up, died from causes other than lymphoma, or were still alive at the last follow-up.

All samples from patients were collected with informed client consent, and all procedures were conducted with relevant guidelines. For the control dogs, canine blood samples from active patients without any neoplastic purposes or healthy volunteers at the NTUVH were collected or purchased with owner consent. Dogs with a client questionnaire and physical exam by the attending veterinarian were determined as controls. This study was approved by the Institutional Animal Care and Use Committee of National Taiwan University, Taipei, Taiwan (protocol code: IACUC No. NTU107-EL-00200).

### 2.2 Immunophenotyping by FC

The peripheral blood lymphocytes (PBLs) were isolated from the whole blood. The whole blood was collected in blood collection tubes, and red blood cells (RBCs) were lysed by adding RBC lysis buffer (Thermo Fisher Scientific, catalog No. 00-4,333-57, Waltham, MA, USA) for 5 min at room temperature. FNAs of the canine lymphoma samples were collected and rinsed several times with PBS buffer. Then the PBS buffer was added to the suspensions and centrifuged at 800 g for 10 min at 4 °C. After the supernatants were removed, the pellets were washed twice with FACS buffer (2% bovine serum albumin in PBS). The cells in PBS suspensions were counted and adjusted to 1×10^6^ cells per 100 μL FACS buffer. The tubes containing cells were incubated with certain fluorochrome-conjugated monoclonal antibodies. As shown in Supplementary Table S1, selected monoclonal anti-CD3, CD21, CD4, and CD8 and their corresponding fluorescein-conjugated isotype antibodies were used for staining surface antigens. Cells were incubated for 30 min at 4 °C. After incubation, the cells were rinsed twice with FACS buffer and resuspended in PBS. Samples were analyzed with an LSR Fortessa Flow Cytometer (BD Biosciences, San Jose, CA, USA). Data were analyzed in FlowJo v10 software (BD Biosciences).

### 2.3 PARR analysis of IgG and TCRγ

PARR assay was performed according to the method published in a previous study (11). Genomic DNA was extracted using the DNeasy blood and tissue kit (Qiagen, catalog no. 69504, Hilden, Germany) according to the manufacturer’s instructions. The primer pairs used are described in Table 1. The DNA concentration was measured with a NanoPhotometer™ (Implen GmbH, Munich, Germany). Then a total amount of 200 ng DNA combined with 1 μL of each primer, buffer, and nuclease-free water was added in a microtube in a volume of 25 μL. The samples were initially heated to 95 °C for 15 min. The cycling profile, namely, 94 °C for 8 s, 60 °C for 10 s, and 72 °C for 15 s, was repeated for 35 cycles. No final extension time was used. The PCR products were analyzed on 10% native polyacrylamide gel electrophoresis with Tris-borate-EDTA buffer. The results were considered positive if one or more dominant and discrete clonal bands were present at irregular intervals. In contrast, polyclonal amplification, diffuse smears, or no band being detected was considered negative results. As a positive control for B-cell lymphoma, CLBL-1 (26), a canine B-cell lymphoma cell line, was used. A canine T-cell lymphoma cell line, UL-1 (27), was employed for T-cell control. Negative controls consisted of PCR reaction mixtures, but the DNA template was excluded. The Qsep 100 DNA capillary electrophoresis system (BiOptic, New Taipei City, Taiwan) was employed for the separation and detection of free nucleic acids and reaction products. Fluorescence detection was facilitated by the use of blue light-emitting diode (LED) lamps as excitation sources. DNA alignment markers, specifically the 20 base pair (LM) and 1,000 base pair (UM), along with size markers ranging from 20 to 1,500 base pairs, were procured from BiOptic (New Taipei City, Taiwan) and stored at −20 °C immediately upon receipt. The interpretation of PARR results was defined as previously described (28).

**Table 1:** Primers used in this study for amplification of Cμ (internal control), IgH major, and TCRγ.

Products	Primer name	Primer sequence (5′ – 3′)	Product size (bp)
Cμ	Sigmf1	TTC CCC CTC ATC ACC TGT GA	130
	Srμ3	GGT TGT TGA TTG CAC TGA GG	
IgH major	CB1	CAG CCT GAC AGC CGA GGA CAC	120
	CB2	TGA GGA GAC GGT GAC CAG GGT	
IgH minor	CB1	CAG CCT GAC AGC CGA GGA CAC	120
	CB3	TGA GGA CAC AAA GAG TGA GG	
TCRγ	DPA	CTG TTG KTG CAG AAR CTG GAG AAG	120
	DPB	AAC CCT GAG AAT TGT GCC AGG AC	
	DPC	GAG TTA CTA TAA TCC TGG TAM CTT CTG	

### 2.4 Statistical analysis

The data are presented as mean ± standard deviation (SD). Statistical analyses were performed in GraphPad Prism v8 and R software (R Foundation for Statistical Computing, Vienna, Austria) utilizing the survival and survminer packages. One-way ANOVA was utilized to determine significant differences among groups. Overall survival time (OST) was estimated using the Kaplan–Meier method, and median survival times were reported alongside their 95% confidence intervals (95% CI). Survival curves between groups were compared using the log–rank (Mantel–Cox) test. Furthermore, Cox proportional hazards regression analysis was conducted to estimate hazard ratios (HR) and their respective 95% CIs to evaluate the impact of clinical variables on survival outcomes. Differences were considered statistically significant at a *p* value of less than 0.05.

## 3 Results

### 3.1 Clinical characteristics

In the current study, 441 cases of canine multicentric lymphoma were diagnosed by cytology and comprehensive evaluations at NTUVH. Of the 441 dogs, 252 were further confirmed as BCL or TCL by immunophenotyping and/or genotyping (Table 2). A total of 234 cases (92.9%) were diagnosed by FC, whereas only a relatively small population was diagnosed by PARR without (*n* = 11, 4.4%) or with FC (*n* = 7, 2.7%). Among the 252 dogs, as shown in Table 3, 169 cases were CD21 + BCL (67.1%) and 83 cases were CD3 + TCL (32.9%). The reproductive status was provided in 238/252 cases in the medical records. The distributions of gender were 39.7% intact male (*n* = 100), 12.3% castrated male (*n* = 31), 28.6% intact female (*n* = 72), 13.9% spayed female (*n* = 35) dogs, and 5.5% (*n* = 14) missing records. The male/female ratio was 1.22 (131:107). The most frequently examined samples for diagnosis were FNA samples (*n* = 201, 79.8%), followed by blood samples (*n* = 39, 15.5%) and others, including cerebrospinal fluid (CSF; *n* = 8, 3.2%) and bone marrow (*n* = 2, 0.8%). Ages were known for 234 dogs; the median age was 10 years (ranging from 1 to 18 years). The mean and mode ages were 9.35 and 11 years, respectively (Supplementary Figure S1). The breed was reported in 182 cases, consisting of 1 giant breed dog (0.4%), 36 large breed dogs (14.3%), 41 medium breed dogs (16.3%), 73 small breed dogs (28.9%), and 31 toy breed dogs (12.3%). In 70 cases (27.8%), however, the breed was not reported. The most prevalent breeds included mixed dogs (*n* = 34, 12.7%), Golden Retrievers (*n* = 30, 11.9%), Miniature Schnauzers (*n* = 14, 5.6%), Miniature Dachshunds (*n* = 12, 4.8%), and Pembroke Welsh Corgis (*n* = 11, 4.4%). The detailed characteristics of the dogs are summarized in Table 3 and Supplementary Table S2.

**Table 2:** Case numbers of BCL and TCL classified by different diagnostic tools.

Diagnostic methods	Final diagnosis	
	BCL (*n* = 169)	TCL (*n* = 83)
FC (*n* = 234)	168	66
PARR (*n* = 11)	0	11*
FC + PARR (*n* = 7)	1	6

*The 11 cases were assigned a presumptive phenotype based solely on the detection of clonal T-cell receptor rearrangement via PARR. FC, flow cytometry; PARR, PCR for antigen receptor rearrangement; BCL, B-cell lymphoma; TCL, T-cell lymphoma.

**Table 3:** Characteristics of animals with lymphoma.

Characteristic	Canine (*n* = 252)
Immunophenotype	
T-cell (CD3)	83 (32.9%)*
B-cell (CD21)	169 (67.1%)
Gender	
Male intact	100 (39.7%)
Male castrated	31 (12.3%)
Female intact	72 (28.6%)
Female spayed	35 (13.9%)
Null	14 (5.5%)
Samples	
Blood	39 (15.5%)
Cerebrospinal fluid	8 (3.2%)
Ascites	1 (0.4%)
Bone marrow	2 (0.8%)
Bronchoalveolar lavage	1 (0.4%)
Fine needle aspiration	201 (79.8%)
Prescapular lymph node	3 (1.2%)
Popliteal lymph node	6 (2.4%)
Mesenteric lymph node	1 (0.4%)
Inguinal lymph node	2 (0.8%)
Axillary lymph node	1 (0.4%)
Intestinal lymph node	2 (0.8%)
Other lymph node	24 (9.5%)
Spleen	2 (0.8%)
Miscellaneous	160 (63.5%)
Pleural effusion	0 (0.0%)
Age (years)	
Mean	9.35 (1–18)
Median	10
Mode	11
Breed	
Giant breeds	1 (0.4%)
Large breeds	36 (14.3%)
Medium breeds	41 (16.3%)
Small breeds	73 (28.9%)
Toy breeds	31 (12.3%)
Null	70 (27.8%)

*These include 11 cases that were assigned a presumptive phenotype based solely on the detection of clonal T-cell receptor rearrangement via PARR.

### 3.2 Flow cytometric analysis

Of 252 dogs with lymphomas, FC analysis was examined in 241 cases, and 7 cases had flow cytometry and PARR results. Furthermore, we also evaluated the subpopulations of immune cells in PBLs from 26 control dogs (Supplementary Table S3); however, due to animal welfare concerns, we did not aspirate the lymph nodes from these dogs. Thus, this study compared the immune cells of lymph nodes to those in a previous study (29).

**Figure 2:**
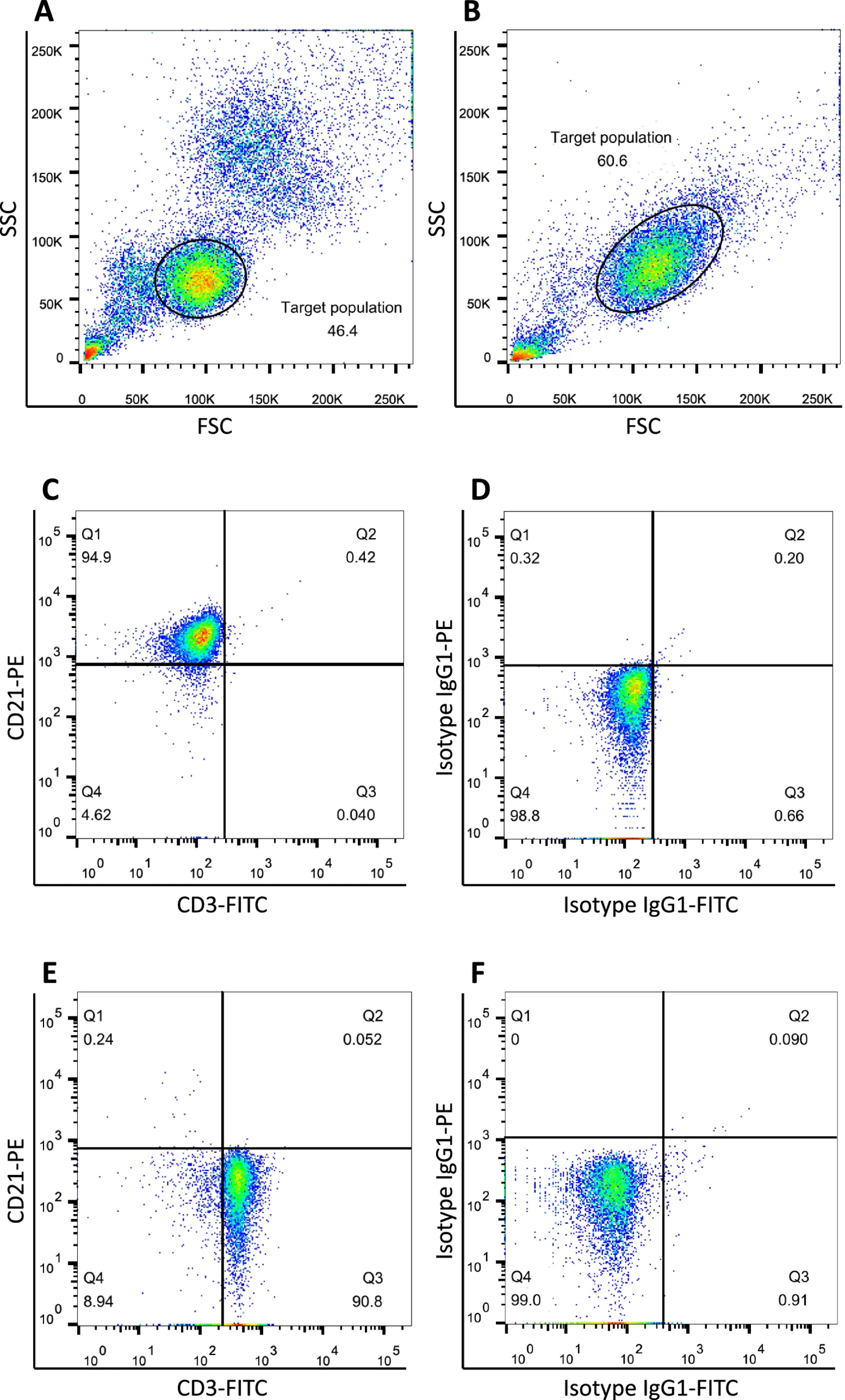
Flow cytometric figures determining subpopulation in canine lymphoma. **(A)** Representative dot plot for forward (FSC) and side scatter (SSC) for evaluation of cell size and granularity in the blood and **(B)** peripheral lymph node of dogs with lymphoma. **(C)** Representative figure of dogs with CD21 + BCL and its **(D)** corresponding isotype control () Representative flow cytometric figure of CD3 + TCL **(E)** and its **(F)** corresponding isotype control.

The populations of B, T, and T subset lymphocytes in PBLs derived from healthy dogs were first evaluated. The representative cell distribution in a normal canine PBL sample is shown in Figure 2A. From the cells gated in the lymphocyte population (“lymphocytes” in Figure 2A), 66.7 ± 12.6% were positive for the common extracellular T-lymphocyte marker CD3. On average, 16.8 ± 8.2% of the gated cells were classified as B-lymphocytes based on labeling with CD21. The CD3 + T-lymphocyte population included 46.7 ± 10.0% CD4 + CD8-T-helper cells, 34.1 ± 10.5% CD4-CD8 + cytotoxic T-cells, and a small population (0.9 ± 0.7%) of CD4 + CD8 + double-positive lymphocytes (Supplementary Figures S2B,C).

Dogs were presented to the NTUVH for high suspicion of lymphoma based on several criteria: enlarged lymph nodes and/or median to large lymphocytes exceeding 50% under cytological evaluation. Then the blood or FNA samples were submitted to FC or PARR analysis. An increased cell population in the blood sample (Figure 2A) and enhanced cell size in the FNA sample (Figure 2B) strengthened the possibility of lymphoma. CD3 versus CD21 plots of gated cells showed a predominant population of CD21 (Figures 2C,D) or CD3 cells (Figures 2E,F). Through this comprehensive analysis, dogs were then phenotypically diagnosed as CD3 + TCL (*n* = 66) or CD21 + BCL (*n* = 168).

### 3.3 PCR for antigen receptor rearrangement

**Figure 3:**
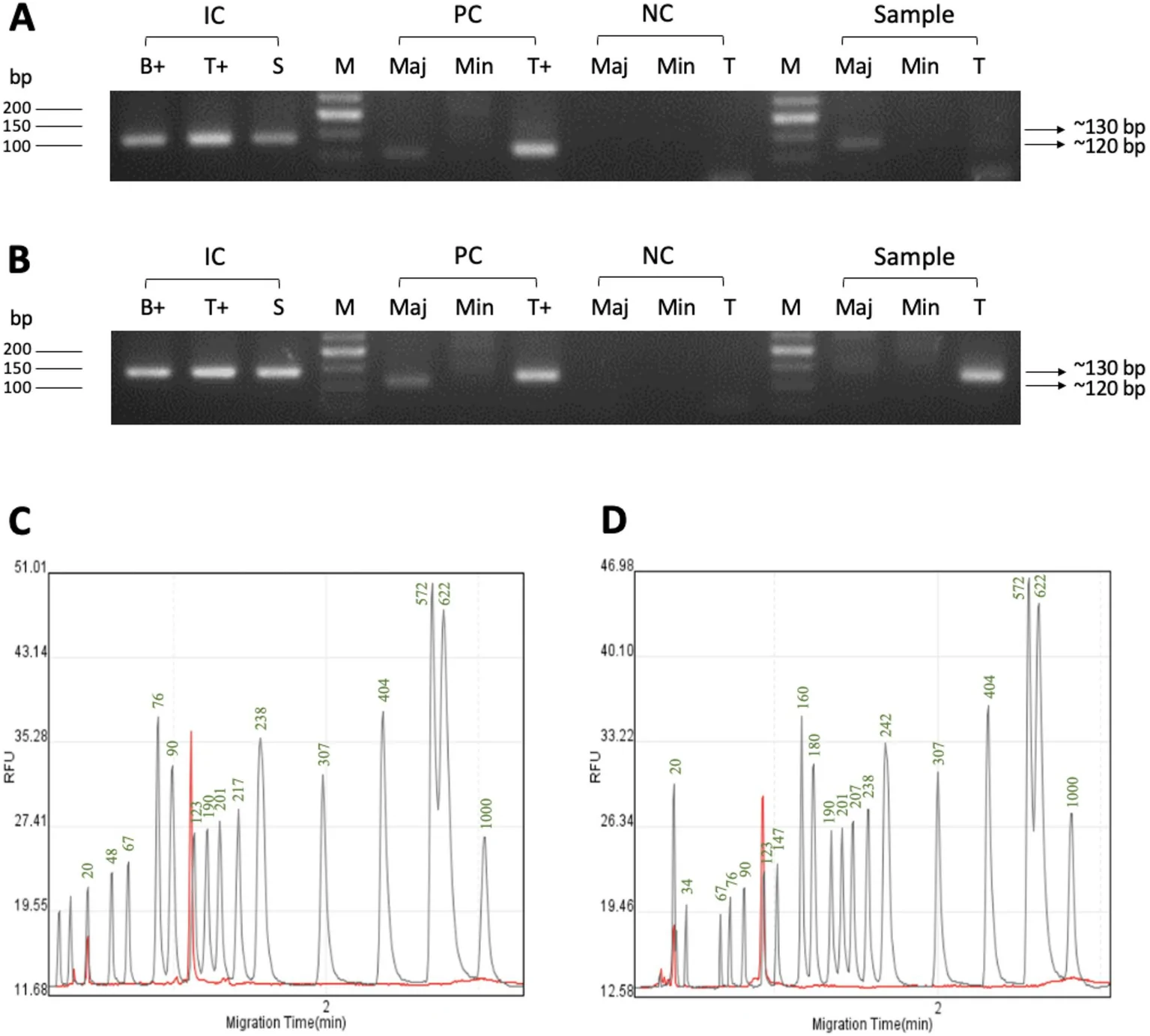
Representative electrophoresis gel figures and typical electropherograms of PARR showing clonal products. For electrophoresis gel figures, clonal products from **(A)** dogs with BCL and **(B)** TCL were shown. The product size was 130 base pairs for Cμ (internal control and product sizes of IgH major, IgH minor, and TCRγ were 120 base pairs). For typical clonal electropherograms, size markers were shown in gray, and the standard markers were labeled in green. **(C)** Representative clonal products from a dog with BCL or TCL **(D)**. Samples were shown in red, and a single tall sharp peak is shown around the anticipated product size. B+: Positive control of B cell lymphoma (CLBL-1 cell line); T+: Positive control of T cell lymphoma (UL-1 cell line); S: Representative sample; M: DNA marker (50 bp); IC: Internal control; PARR: Polymerase chain reaction for antigen receptor rearrangements.

Although 234 cases (92.9%) were diagnosed by FC, only 18 suspected cases of lymphoma were analyzed by PARR, including 11 cases (4.4%) or 7 cases (2.7%) without or with FC results. These results indicated that FC was usually widely used in the clinic when lymphoma was suspected. In the gel figures, these cases had clonal immunoglobulin or TCR rearrangements, supporting the diagnosis of BCL (Figure 3A) or TCL (Figure 3B). The original gel blots are shown in Supplementary Figure S3. In PARR electropherograms, these samples had one dominant peak at the anticipated product size, suggesting that the samples contained a clonal rearrangement (Figures 3C,D). In the 7 cases with both FC and PARR results, the immunophenotypes and the genotypes were consistent. Although the immunophenotype and genotype were correlated in the tested samples, given the small sample size of cases with both FC and PARR results, definitive and broad conclusions regarding phenotype–genotype correlation cannot be established in this study.

### 3.4 Patient outcome

**Figure 4:**
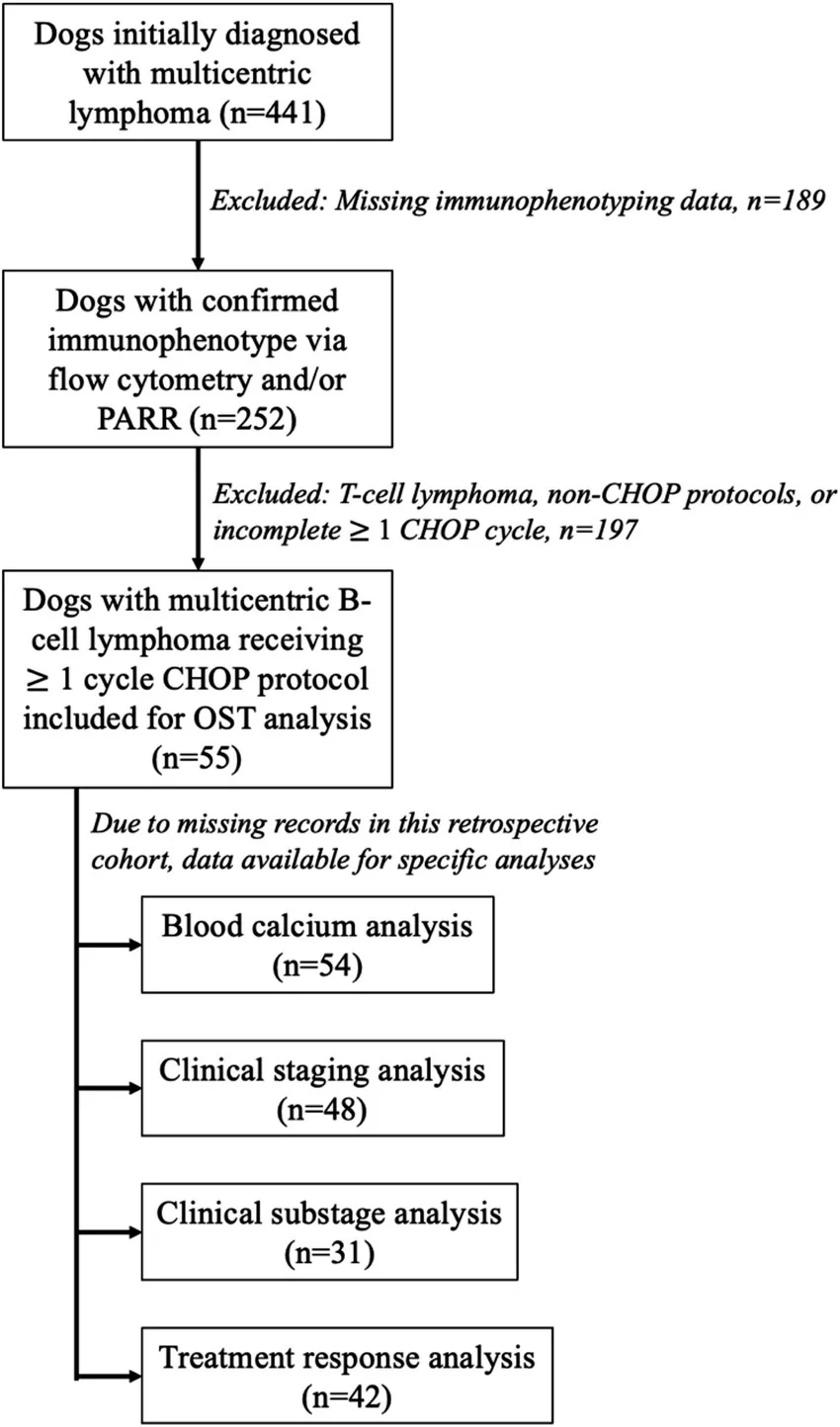
Flowchart of the case selection and study population. A total of 441 dogs were initially diagnosed with multicentric lymphoma. Among them, 252 cases underwent immunophenotyping via flow cytometry and/or PARR, identifying 169 cases of MBCL and 83 cases of TCL. For the OST analysis, a specific cohort of 55 dogs with confirmed MBCL that completed at least one full cycle of CHOP chemotherapy was selected. Due to the retrospective nature of this study and missing medical records, the final number of cases available for specific subgroup analyses varied: Blood calcium concentration (*n* = 54), clinical staging (*n* = 48), clinical substage (*n* = 31), and treatment response (*n* = 42). MBCL, multicentric B-cell lymphoma; OST, overall survival time; PARR, polymerase chain reaction for antigen receptor rearrangements; TCL, T-cell lymphoma.

The above sections reported the diagnostic methods and immune cells of lymphoma at NTUVH. Then, the authors next clarified the correlation between clinical parameters and OST in these selected dogs (*n* = 55). These 55 dogs were confirmed to have MBCL and had received at least one full cycle of the standard CHOP chemotherapy protocol (Figure 4). Due to the retrospective nature of this study, certain clinical parameters were not uniformly recorded for all 55 patients. Consequently, subgroup survival analyses were performed based on the available data for each variable: blood calcium concentrations were available for 54 dogs, clinical staging for 48 dogs, clinical substage for 31 dogs, and definitive treatment response (CR/PR/SD/PD) could be thoroughly tracked for 42 dogs. The correlations between several clinical parameters and OST in dogs were further reported.

**Figure 5:**
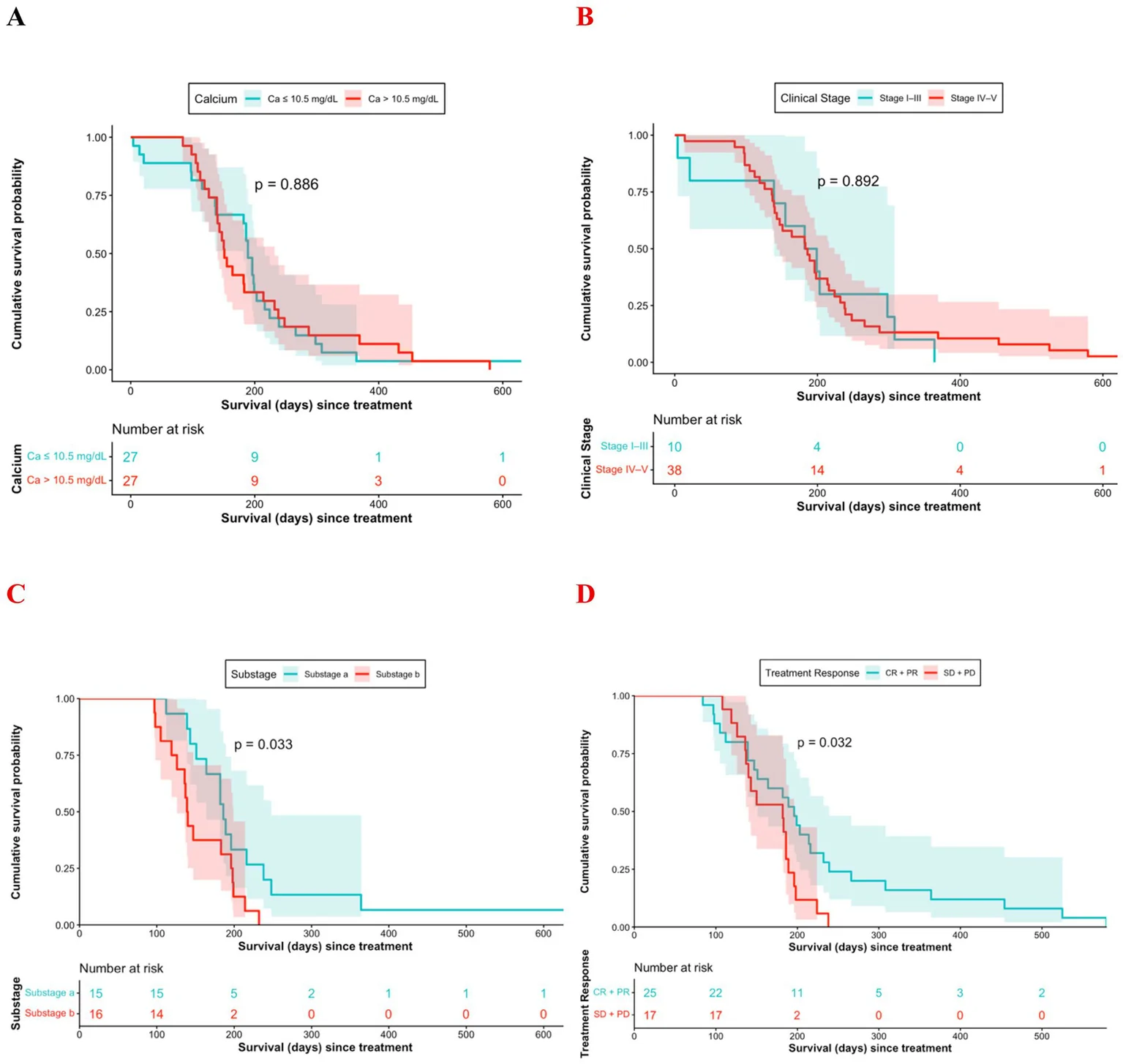
Kaplan–Meier curve depicting OST of dogs with MBCL stratified by different clinical parameters. All dogs completed at least one full cycle of CHOP chemotherapy. **(A)** Kaplan–Meier curves illustrating the OST of 54 lymphoma patients that were stratified by the median blood calcium concentration (10.5 mg/dL) at tumor diagnosis (*p* = 0.886). **(B)** Kaplan–Meier curves for OST of 48 lymphoma patients that were stratified by clinical stages (*p* = 0.892). **(C)** Kaplan–Meier curves for OST of 31 lymphoma patients that were stratified by substage (*p* = 0.033). **(D)** Kaplan–Meier curves for OST of 42 lymphoma patients that were stratified by treatment responses (*p* = 0.032). Tick marks represent dogs that were alive at last follow-up.

MST was not significantly shorter in dogs with higher serum calcium levels (median: 151 days, 95% CI: 140–232 days) than in those with lower serum calcium levels (Figure 5A, median: 189 days, 95% CI: 182–216 days). Furthermore, Cox proportional hazards analysis indicated that higher serum calcium level relative to the cohort median was not significantly associated with an increased risk of death (HR = 1.04, 95% CI: 0.60–1.79, *p* = 0.894). Furthermore, no significant difference in survival was found between dogs with clinical stages I–III MBCL (median: 190 days, 95% CI: 139–NR) and those with clinical stages IV–V (Figure 5B, median: 184 days, 95% CI: 143–224 days). Cox proportional hazards analysis also revealed that an advanced clinical stage (IV–V) was not significantly associated with an increased risk of death compared to stages I–III (HR = 0.95, 95% CI: 0.47–1.94, *p* = 0.891). Dogs presenting with clinical signs (substage b) had a significantly shorter overall survival time (median: 140 days, 95% CI: 126–199 days) compared to those without clinical signs (substage a; median: 186 days, 95% CI: 164–248 days; Figure 5C). Furthermore, Cox proportional hazards analysis demonstrated that the presence of clinical signs (substage b) was significantly associated with a higher risk of death compared to asymptomatic dogs (HR = 2.27, 95% CI: 1.04–4.96, *p* = 0.040). Dogs that achieved a complete or partial response (CR or PR) to CHOP therapy had a significantly longer overall survival time (median: 196 days, 95% CI: 151–239 days) compared to dogs with stable or progressive disease (SD or PD; median: 182 days, 95% CI: 140–196 days; Figure 5D). Furthermore, Cox proportional hazards analysis demonstrated that dogs failing to achieve an objective response (SD or PD) had a significantly higher risk of death compared to responders (HR = 2.09, 95% CI: 1.06–4.16, *p* = 0.035). These results suggested that dogs with obvious clinical symptoms and worse treatment responses tended to have shorter OST.

## 4 Discussion

This study retrospectively reviewed the profiles of dogs with lymphoma in Taiwan. The OSTs of dogs with MBCL divided by clinical variables and treatment responses are also reported here. To the authors’ knowledge, this is the first retrospective study to present such information on canine MBCL in Taiwan.

We found in this study that FC analysis is more commonly used than PARR for discriminating BCL or TCL. Of 252 cases with lymphoma, 241 cases (95.6%) were characterized by FC or FC combined with PARR, while only 11 cases underwent PARR analysis (4.4%). These findings might be explained by several reasons. FC analysis has higher sensitivity than PARR analysis (4). FC can detect aberrant expression of B and T cells via multiple panels (3, 7), whereas PARR provides limited information, only showing the clonality in a population of lymphoid cells. However, PARR can be performed when fresh samples are not available. This assay can be conducted using samples from dead cells, effusions, and formalin-fixed paraffin-embedded tissues (4, 30), to which FC analysis is not applicable. Furthermore, when surface antigens are down-regulated, PARR will define a lineage more accurately than FC (28). PARR is the only assay that can detect the true clonality of a population of lymphoid cells and should be suggested as the first-line assay in ambiguous cases (4). While PARR is valuable for determining clonality, canine lymphomas can exhibit cross-lineage rearrangements (31). Consequently, phenotype assignment based solely on PARR carries a risk of misclassification bias. A methodological limitation in our data is the inclusion of 11 TCL cases classified solely by PARR (Table 2). Therefore, all PARR-only cases were excluded from our primary survival analyses, which focused exclusively on FC-confirmed B-cell lymphomas, to ensure scientific rigor.

OST was longer in dogs with BCL than in those with TCL in this study (data not shown) when we roughly grouped these cases stratified by FC and/or PARR results. This result was expected, as several previous studies have proposed that the presence of B-cell disease is a positive prognostic factor with medical intervention (21, 32). The increased OST in BCL patients resulted from multiple factors. One was that more rapid acquisition of drug resistance occurs in dogs with TCL (5, 33). Among the dogs with BCL in this study, the resulting T cells in their peripheral blood were still identified. In contrast, neoplastic T cells in TCL were dysfunctional, lacking their normal anti-tumor immunity (34, 35). Thus, we speculated that this might be another of the reasons why the OST of dogs with BCL was higher than that of those with TCL. However, there are numerous types of BCL or TCL. The authors believed that although we identified that OST was longer in dogs with BCL than in those with TCL, these results remained controversial. Therefore, we focused on discussing the OST of dogs with MBCL.

In dogs with MBCL, increased OST was also found in patients with substage a (Figure 5C) and responses to treatment (Figure 5D). While our analysis revealed a statistically significant difference in overall survival time based on treatment response, the absolute difference in median survival between responders (196 days) and non-responders (182 days) was only 14 days. Given the relatively small sample size in our survival cohort, this narrow median difference should be interpreted with caution from a clinical perspective. However, it is important to note that median survival represents only a single point on the survival curve. Our Cox proportional hazards analysis provides a more comprehensive assessment, demonstrating that dogs failing to achieve an objective response had a consistently higher overall risk of mortality throughout the disease course (HR = 2.09). This suggests that initial treatment response was statistically associated with OST in this retrospective cohort; however, it should not be interpreted as a stand-alone surrogate for durable remission or long-term prognosis. By contrast, animals with increased serum calcium and infiltrations of neoplastic cells in the liver or spleen (stage IV) and the BM or CNS (stage V) had no difference in OST compared with those without these findings (Figures 5A,B). One possible explanation is that, compared with these laboratory examinations, the clinical signs and treatment responses had greater impacts on survival. In other words, a dog might have a longer survival with responses to treatment and the absence of obvious clinical signs despite higher serum calcium levels or an advanced clinical stage. Second, TCL demonstrates a significantly stronger correlation with increased serum calcium than BCL. A study comparing 13 TCL and 22 BCL dogs found hypercalcemia in 31% of TCL cases versus only 4.5% of BCL cases (1/22) (36). Although the current study did not distinguish the detailed subtypes of TCL or BCL, our results might echo these findings. However, the authors believe that it would be prudent to clarify these issues in further studies.

This study had some limitations. The standard phenotypes in multicolor immunophenotyping have been widely proposed for identifying several subsets of neoplastic cells (3, 4, 7–9). However, this retrospective study restricted our explorations. Further prospective studies using these published panels to characterize different types of BCL or TCL should be conducted. Cases with lymphoma were stratified by BCL or TCL based on CD21 or CD3 positivity (or PARR in a few cases). Standard immunophenotyping (CD3 positivity for TCL or CD21 positivity for BCL) failed to distinguish the less aggressive lymphomas or more advanced malignancies in the same lymphoma phenotype. In addition, histopathological grade, mitotic index, Ki-67, and immunohistochemical or molecular markers such as MUM1, BCL-6, and CD34 were not consistently available. Therefore, this study could not evaluate the biological heterogeneity within MBCL or determine whether clinical signs and treatment response remained associated with OST after adjustment for tumor biology. Furthermore, the heterogeneity of the treatment protocols and missing medical records are other limitations. Patients received 15-, 19-, or 25-week CHOP protocols based on attending clinician preferences, owner financial constraints, and patient compliance. The exact intended protocol duration, including allocation to the 15-, 19-, or 25-week CHOP protocols, could not be reliably reconstructed for all dogs because of incomplete retrospective medical records. These variables that could significantly impact survival, such as dose reductions, treatment interruptions, and the exact number of completed cycles, were inconsistently recorded in this retrospective cohort and could not be robustly incorporated into a multivariable analysis. Therefore, the statistical difference in OST may be affected. Detailed case-level information regarding nodal and extranodal involvement, tumor burden, and longitudinal treatment response was not consistently recorded for all dogs. Therefore, robust adjusted analyses incorporating tumor burden, gastrointestinal or visceral involvement, age, breed, and treatment duration could not be performed. These unmeasured or incompletely recorded confounders may have influenced the observed associations between clinical signs, treatment response, and OST. Future prospective studies are required to meticulously control for these therapeutic variables. Last, in this study, 11 cases were classified as TCL based solely on PARR. These cases represent a presumptive diagnosis rather than a definitive one (Table 2). Relying exclusively on genotypic clonality for lineage assignment introduces a potential immunophenotypic misclassification bias, primarily due to the risk of cross-lineage rearrangements in canine lymphoma. Although this bias does not impact the OST analysis shown in Figure 5, it highlights a critical diagnostic challenge in retrospective veterinary studies. Future prospective studies should mandate definitive phenotypic confirmation utilizing FC and/or IHC for all enrolled cases, reserving PARR as an adjunctive assay for assessing clonality and detecting minimal residual disease, rather than as a standalone tool for lineage assignment.

In conclusion, canine patients presenting with clinical signs demonstrated a significantly reduced OST compared to asymptomatic counterparts. Dogs exhibiting complete or partial responses to CHOP therapy achieved prolonged OST relative to non-responders. This study constitutes the first epidemiological and immunophenotypic characterization of canine MBCL in Taiwan, though further investigations are warranted to comprehensively elucidate the molecular and/or clinical signatures of canine lymphoma in this region.

## Data Availability

The original contributions presented in the study are included in the article/Supplementary material, further inquiries can be directed to the corresponding author.
