# Supplementary material for: Correlation between clinical variables and overall survival time in dogs with multicentric B-cell lymphoma: a retrospective study in Taiwan
Source: Front Vet Sci. 2026 Aug 17;13:1870235. doi: 10.3389/fvets.2026.1870235 (PMC13526614; doi:10.3389/fvets.2026.1870235)
Supplement: Supplementary file 1 [file Supplementary_file_1.docx]

Supplementary Material

**Supplementary Figure 1.** Age distribution of dogs diagnosed as lymphomas. The mean, median and mode for canines were 9.35, 10, and 11 years old.

**Supplementary Figure 2.** Flow cytometric phenotypes determined in normal blood of 26 dogs. (**A**) Representative dot plot for forward (FSC) and side scatter (SSC) for evaluation of cell size and granularity. (**B**) Top row: representative dot plots showing cells labeled with isotype control antibodies. The figure for CD4 and CD8 was determined by the CD3- cells. Bottom row: representative dot plots showing cells labeling positive with antibodies to CD3, CD21, CD4, and CD8. The CD4 and CD8 analyses were gated on the CD3+ T cells. (**C**) Statistical analysis of determined lymphocyte subpopulations in normal blood of dogs. The data are expressed as mean ± SD.

**A**

**B**

**Supplementary Figure 3.** Representative polymerase chain reaction for antigen receptor rearrangements (PARR) electrophoresis; original gel figures.

**Supplementary Table 1.** Antibodies used in this study for detection of B or T cells and negative control.

| **Antibody name** | **Specificity** | **Clone** | **Catalog** |
| --- | --- | --- | --- |
| Mouse anti Dog CD3 FITC | Dog CD3 | CA17.2A12 | Biorad, MCA1774F |
| Mouse anti Dog CD21 RPE | Dog CD21 | CA2.1D6 | Biorad, MCA1781PE |
| Rat anti Dog CD4 RPE-Cy7 | Dog CD4 | YKIX302.9 | Biorad, MCA1038PECY7 |
| Rat anti Dog CD8 Alexa Fluor® 647 | Dog CD8 | YCATE55.9 | Biorad, MCA1039A647 |
| Mouse IgG1 negative control: FITC | Mouse IgG1 |  | Biorad, MCA928F |
| Mouse IgG1 negative control: RPE | Mouse IgG1 |  | Biorad, MCA928PE |
| Rat IgG2a Negative Control: RPE-Cy7 | Rat IgG2a |  | Biorad, MCA6005PECY7 |
| Rat IgG1 Negative Control: Alexa Fluor® 647 | Rat IgG1 |  | Biorad, MCA6004A647 |

**Supplementary Table 2.** Breeds of neoplastic dogs categorized by the American Kennel Club (AKC).

|  | **Canine (n=252)** | **Breed Weight Chart (pounds)** |
| --- | --- | --- |
| **Breed** |  |  |
| **Giant breeds** | **1 (0.4%)** | **75 to 120 pounds** |
| Bernese Mountain Dog | 1 (0.4%) | 70–115 |
| **Large breeds** | **36 (14.3%)** | **55 to 85 pounds** |
| Old English Sheepdogs | 2 (0.8%) | 60–100 |
| Retrievers (Golden) | 30 (11.9%) | 55–75 |
| Retrievers (Labrador) | 4 (1.6%) | 55–80 |
| **Medium breeds** | **41 (16.3%)** | **35 to 65 pounds** |
| Border Collies | 1 (0.4%) | 30–55 |
| Bulldogs | 1 (0.4%) | 40–50 |
| Finnish Spitz | 1 (0.4%) | 20–33 |
| Mixed | 34 (12.7%) | Undefined |
| Spaniels (English Cocker) | 1 (0.4%) | 28–34 |
| Siberian Huskies | 3 (1.2%) | 35–60 |
| **Small breeds** | **73 (28.9%)** | **7 to 35 pounds** |
| Beagles | 5 (2.0%) | 20–30 |
| Cavalier King Charles Spaniels | 1 (0.4%) | 13–18 |
| Dandie Dinmont terriers | 1 (0.4%) | 18–24 |
| French Bulldogs | 3 (1.2%) | Less than 28 pounds |
| Fox Terriers | 1 (0.4%) | 6–18 |
| Miniature Dachshunds | 12 (4.8%) | Less than 11 pounds |
| Miniature Pinschers | 1 (0.4%) | 8–10 |
| Miniature Poodles | 5 (2.0%) | 10–15 |
| Miniature Schnauzers | 14 (5.6%) | 11–20 |
| Pembroke Welsh Corgis | 11 (4.4%) | Less than 28 to 30 |
| Pugs | 2 (0.8%) | 14–18 |
| Shiba Inu | 2 (0.8%) | 17–23 |
| Shih Tzu | 9 (3.6%) | 9–16 |
| West Highland White Terriers | 5 (2.0%) | 15–20 |
| Whippets | 1 (0.4%) | 25–40 |
| **Toy breeds** | **31 (12.3%)** | **2 to 9 pounds** |
| Chihuahua | 9 (3.6%) | Less than 6 pounds |
| Maltese | 18 (7.1%) | Less than 7 pounds |
| Pomeranian | 1 (0.4%) | 3–7 |
| Yorkshire Terriers | 3 (1.2%) | 7 |
| **Null** | **70 (27.8%)** |  |

*The breed weight chart and breeds were defined by the AKC.

**Supplementary Table 3.** Description of 26 healthy dogs enrolled in the study. M, intact male; MC, castrated male; F, intact female; FSp, spayed female.

| No. | Reproductive status | Breed | Age (years) | Weight (kg) | CD3+^1^ | CD21+^1^ | CD3-CD21-^1^ | CD4+^2^ | CD8+^2^ | CD4-CD8-^2^ | CD4+  CD8+^2^ |
| --- | --- | --- | --- | --- | --- | --- | --- | --- | --- | --- | --- |
| 1 | M | Beagle | 4.5 | 12 | 85.30 | 8.50 | 5.10 | 43.10 | 41.60 | 14.70 | 0.70 |
| 2 | M | Beagle | 3.0 | 10 | 69.10 | 21.20 | 9.20 | 37.30 | 21.70 | 40.80 | 0.20 |
| 3 | MC | Beagle | 4.0 | 12 | 87.90 | 5.40 | 5.90 | 33.50 | 55.50 | 10.90 | 1.00 |
| 4 | F | Beagle | 1.0 | 10 | 61.80 | 26.30 | 11.80 | 48.40 | 39.70 | 11.70 | 0.20 |
| 5 | FSp | Beagle | 5.5 | 9 | 78.80 | 13.30 | 8.00 | 57.30 | 24.20 | 18.00 | 0.40 |
| 6 | FSp | Beagle | 6.5 | 8 | 46.84 | 14.47 | 37.89 | 45.64 | 44.34 | 9.24 | 0.77 |
| 7 | M | Border Collie | 8.5 | 20 | 73.20 | 15.45 | 9.51 | 46.31 | 33.95 | 18.16 | 1.58 |
| 8 | M | Border Collie | 4.0 | 19 | 78.89 | 9.76 | 10.59 | 59.82 | 25.48 | 14.35 | 0.35 |
| 9 | MC | Border Collie | 2.0 | 22 | 58.15 | 23.90 | 14.73 | 59.42 | 23.27 | 16.55 | 0.76 |
| 10 | FSp | Border Collie | 5.5 | 16 | 61.39 | 13.35 | 22.07 | 62.68 | 22.58 | 14.10 | 0.63 |
| 11 | M | Formosan | 1.5 | 51 | 78.37 | 10.07 | 10.08 | 55.51 | 30.72 | 13.29 | 0.49 |
| 12 | MC | Formosan | 2.5 | 54 | 71.27 | 10.74 | 16.22 | 50.49 | 27.81 | 20.80 | 0.90 |
| 13 | MC | Formosan | 1.5 | 53 | 69.77 | 14.04 | 14.62 | 43.64 | 40.02 | 15.72 | 0.63 |
| 14 | MC | Formosan | 2.5 | 56 | 59.97 | 19.67 | 17.63 | 37.55 | 32.77 | 27.81 | 1.80 |
| 15 | MC | Formosan | 1.5 | 51 | 77.05 | 3.80 | 17.56 | 35.16 | 51.27 | 10.05 | 3.51 |
| 16 | F | Formosan | 4.5 | 48 | 71.30 | 5.61 | 20.66 | 33.77 | 57.70 | 6.45 | 2.08 |
| 17 | F | Formosan | 3.0 | 49 | 48.17 | 15.06 | 36.25 | 45.70 | 42.91 | 11.00 | 0.39 |
| 18 | F | Formosan | 4.0 | 51 | 39.91 | 24.65 | 34.57 | 44.60 | 40.90 | 14.95 | 0.27 |
| 19 | F | Formosan | 3.5 | 47 | 61.43 | 15.81 | 21.91 | 45.23 | 32.96 | 20.69 | 1.11 |
| 20 | FSp | Formosan | 3.5 | 50 | 86.12 | 8.85 | 4.72 | 54.67 | 30.35 | 14.51 | 0.47 |
| 21 | FSp | Formosan | 2.0 | 43 | 67.60 | 26.40 | 4.80 | 47.70 | 32.10 | 19.50 | 0.70 |
| 22 | FSp | Formosan | 7.0 | 45 | 54.80 | 18.20 | 26.00 | 44.60 | 22.40 | 31.20 | 1.80 |
| 23 | FSp | Irish Setter | 1.5 | 30 | 65.10 | 21.20 | 13.00 | 24.90 | 37.30 | 36.90 | 1.00 |
| 24 | M | Irish Setter | 5.5 | 32 | 47.50 | 35.90 | 16.00 | 56.70 | 23.70 | 16.00 | 0.60 |
| 25 | MC | Mixed | 3.0 | 20 | 69.20 | 24.10 | 5.80 | 37.20 | 26.40 | 35.50 | 0.90 |
| 26 | FSp | Mixed | 2.5 | 15 | 64.30 | 29.90 | 0.50 | 63.70 | 23.90 | 11.70 | 0.60 |

^1^ Subset of the T cells gated on the lymphocyte population.

^2^ Subset of the T cells gated on the CD3+ T lymphocyte population.
